# Continuing education interventions about person-centered care targeted for nurses in older people long-term care: a systematic review

**DOI:** 10.1186/s12912-021-00585-4

**Published:** 2021-04-28

**Authors:** Mari Pakkonen, Minna Stolt, Andreas Charalambous, Riitta Suhonen

**Affiliations:** 1 Department of Nursing Science, University of Turku, Turku, Finland; 2 Department of Nursing Science, Cyprus University of Technology, Limassol, Cyprus; 3 Turku University Hospital, Turku, Finland; 4 City of Turku, Welfare Division, Turku, Finland

**Keywords:** Person-centered care, Continuing education, Older people, Long-term care, Intervention, Review

## Abstract

**Background:**

Person-Centered Care is often seen as an indicator of quality of care. However, it is not known whether and to what extent person-centered care can be enhanced by continuing education interventions in older people’s long-term care settings. This systematic review aimed to analyze and synthesize the existing research literature about person-centered care-based continuing educational interventions for nurses working in long-term care settings for older people.

**Methods:**

Five databases were searched 6/2019 and updated 7/2020; PubMed (Medline), CINAHL, PsycINFO, Cochrane and Eric using the keywords person-centered car* OR person-centred car * OR patient-centered car* OR client-centered car* OR tailored car* OR resident-centered car* OR individualized car* AND older* OR elder* OR old person* AND Long-Term Care OR Nursing home OR 24-h treatment OR long-term treatment. Twenty-seven full texts from 2587 initially retrieved citations were included.

**Results:**

The continuing educational interventions found were divided into five themes: person-centered interventions focusing on medication; interaction and caring culture; nurses’ job satisfaction; nursing activities; and older people’s quality of life. The perspective of older people and their next of kin about the influence of continuing education interventions were largely absent. The background theories about interventions, the measurements taken, and the clarity around the building blocks of the continuing-care interventions need further empirical verification. The pedagogical methods used were mainly quite behavioristic mostly lectures and seminars.

**Conclusion:**

Most of person-centered care continuing education interventions are effective. Still more empirical research-based continuing education interventions are needed that include learner-centered pedagogical methods, with measurable outcomes that consider the opinions of older people and their next of kin. Continuing educational interventions for nurses need to be further developed to strengthen nurse’s competence in person-centered care, job satisfaction and for better quality of care.

## Background

The requirements for quality in the nursing care of older people has been written in most countries into: legislation [1], national guidelines [2, 3] and policy papers [4]. The requirements are also written into international ethical guidelines [5, 6] and e.g. in Finland’s legislation [7, 8]. (Ministry of Social Affairs and Health, 2018; Finlex, 980/2012). Person-centered care is often used as a quality indicator and is of ethical value [2–8].

The conceptual roots of person-centered care are in theories based on the philosophy of humanism, which includes personhood and well-being theory [9], personality theory [10] and nursing theories [11, 12]. In the nursing literature, the concept of person-centered care is often used as a synonym for other terms, such as individualized care, patient-centered care, client-centered care, and personalized care [13]. However, these concepts have differences. The concept of individualized care considers an individual as a biopsychosocial integral whole, focuses on individual differences, preferences, and the values of individuals. Individualized care includes the patient’s clinical situation, personal life situation and decisional control [11]. Person- and patient-centered care concepts have different goals. Patient-centered care considers the patient as the center and prioritizes functional life, whilst the person-centered care takes a wider stance prioritizing the whole life, including the interactions with others and the achievement of a meaningful life [14]. The way in which people are cared for often defines which concept is used in practice, for example, if person is given the status of a client or patient [13]. The core of each of the care concepts is human autonomy and respect for dignity [11, 13–15]. Person-centered care asserts its strong place in the care of older people because it is associated with the quality of life [16] and experiences of the quality of care [17] with a whole life orientation including, but not limited to a health problem orientation [14].

Continuing Education been defined in terms of continuing professional development, in-service training, or further training [18]. Usually the purpose of continuing education, which takes place after formal nurse registration education, is to develop nurses’ competence [19]. In different hospital settings, individual nurse’s competence consists of personal skills, abilities, and knowledge [20]. The collective competence of a group or team of nurses has a broader base and is greater than that of the sum of the individuals’ competence, facilitating the growth and development of individuals with varying competence within the team [21]. Organizational culture has been found to affect the quality of care delivery by nursing teams, both positively and negatively [22]. Therefore, the promotion of competence through in long-term care is important, not only for individual nurses but also the collective competence of the team.

Young people’s perceptions of the nursing profession are quite negative [23]. Nurses (Registered Nurses, Practical Nurses and Nursing Assistants) have reported a high level of job strain in nursing homes where the many residents suffer from dementia [24]. Nurses have and suggested there is poor support from the organization managers for the quality of their work in long-term care [25]. The use of person-centered care may mitigate these issues by reducing stress, burnout, and improving job satisfaction among dementia care nurses, though the research in this area is currently weak making firm conclusions difficult [26]. Person-centered care interventions that have a positive influence in older people’s long-term care discussed in the literature include: environmental changes; interaction relationships; relationships with managers; the empowerment of nurses; staff-resident relationships and the care culture so that care is more about the individual [27]. Psychosocial interventions in person-centered care of older people in long-term care include elements such as: communication training; emotional response support; dementia care mapping; retaining abilities; sensory strategies; integrity-promoting care; organizational level changes; and others not categorized by researchers [28]. The outcomes of these interventions seem to support the use of person-centered care approach although generalizability of the results is limited due to the complexity of the interventions [27, 28]. However, there seems to be little useful evidence about the effects of person-centered continuing education interventions on nurses working with older people in long-term care and even less from the perspective older people and their next of kin. Given the importance of the topic, more needs to be known about the efficacy of person-centered care continuing education interventions, especially as the older people population increases [29] and older people are expected to live longer, many with long-term health conditions [30].

The aim of this review was to analyze and synthesize the existing research literature about person-centered care-based continuing educational interventions for nurses working in long-term care settings for older people. The goal was to increase the understanding of the current pedagogical methods and results of continuing education interventions from the perspective of nurses, older people and their next of kin.

## Methods

This study is a systematic review of the empirical research literature after Harris [31] focused on person-centered care-based continuing education interventions for nurses working in long-term care settings for older people. The review was conducted following internationally recommended scientific practice in every phase [32] and reported according to Preferring Reporting Items for Systematic Reviews and Meta-Analyses PRISMA [33].

### Search strategy

A systematic search from five relevant databases was conducted on 06/2019 and updated on 7/2020, published articles written in English without any time limit: PubMed (Medline), CINAHL, PsycINFO, Cochrane and ERIC using keywords and Boolean operators. The search phrase PubMed (Medline) was: (“Patient-Centered Care”[Mesh] OR person-centered car* OR person-centred car* OR “person centered care” OR “person centred care” OR “patient centered care” OR “patient centred care” OR patient-centered car* OR “patient-centred care” OR “client centered care” OR “client centred care” OR tailored car* OR “resident centered care” OR “resident centred care” OR “resident-centred care” OR “resident-centered care” OR individualized car* OR individualized car*) AND (“Frail Elderly”[Mesh] OR “Aged”[Mesh] OR “Aged, 80 and over”[Mesh] OR “Senior Centers”[Mesh] OR older* OR elder* OR aged OR senior* OR resident* OR old people* OR old person*) AND (“Insurance, Long-Term Care”[Mesh] OR “Long-Term Care”[Mesh] OR “Nursing Homes”[Mesh] OR “After-Hours Care”[Mesh] OR “Conservative Treatment”[Mesh] OR long-term car* OR LTC OR nursing home* OR 24-h treatment* OR 24-h car* OR enhanced treatment* OR enhanced car* OR long-term treatment*).

### Study selection

Studies were included in the review if they were: (1) experimental study designs; RCTs; controlled clinical trials (CCTs); quasi-experimental and pre-posttest studies with or without control groups. (2) intervention studies with person-centered care elements; (3) studies focused on continuing education interventions for nurses working in long-term settings for older people; and (4) peer-reviewed research studies published in the English language. Studies were excluded if they were (1) implementation studies or feasibility studies not assessing any outcomes and (2) interventions other than person-centered care-based continuing educational interventions for nurses working in long-term care settings for older people.

**Fig. 1:**
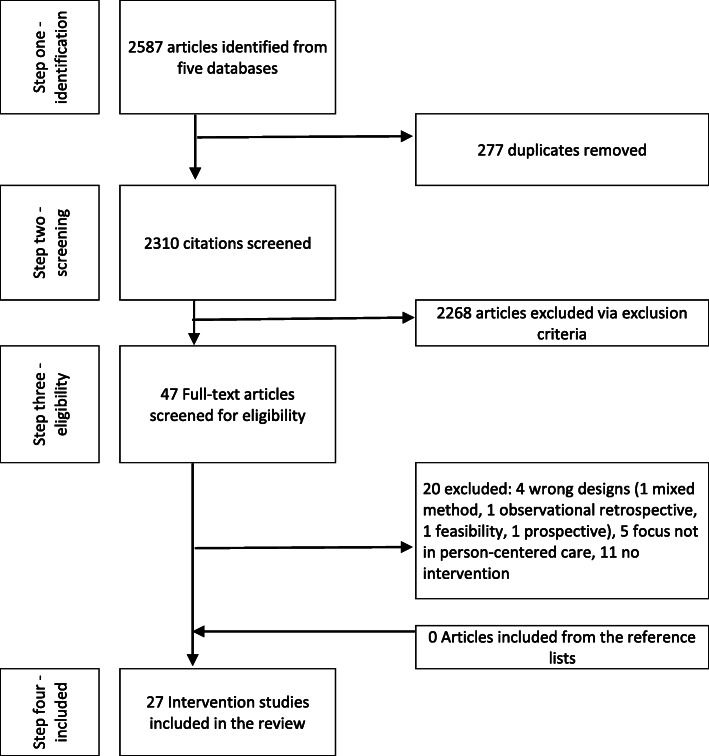
Retrieval of the studies

Retrieval of the studies was conducted in four steps [33] (Fig. 1). The initial search of the databases retrieved 2587 citations. After removing duplicate studies (*n* = 277), in the second step, three researchers (MP, MS and RS) screened the titles and abstracts to identify eligible records for full text analysis removing 2310 scripts. In the third step the remaining 47 papers were analyzed against the inclusion and exclusion criteria. During this analysis researchers eliminated 20 papers by consensus leaving 27 papers for further analysis. A manual search of the reference lists of the 27 included studies identified no further relevant studies.

### Quality appraisal

The quality of the 27 included papers was assessed by Joanna Briggs Institute [34] checklist for quasi-experimental studies. The aim of the appraisals was to assess methodological quality of studies considering bias in designs, research conduct and the analyses with nine appraisal questions and a maximum score of 9. The quality appraisal was recorded but not used as part of the inclusion criteria.

### Data analysis

Data were collected in tabular format: the author(s); year of publication; country of origin; design; aim of study; sample; participants; data collection instruments; and methods. The contents, pedagogical methods and outcomes from the perspective of Nurses (N), Next of kin (NK) and Older People (OP) were also collected within the same tabular format. The outcomes of the interventions, positive or no effect, were analyzed using a two-step categorization (+ = a positive effect of the intervention, 0 = no effect of the intervention). As the outcome variables differed from each other it was not possible to calculate meta-analysis. Data were analyzed using conventional content analysis [35]. The original expressions used by authors in their articles were used to reduce interpretation. The analysis was performed by one researcher and the results were confirmed in the research team before tabulation.

## Results

### Characteristics of the studies

The studies (Table 1) were carried out mainly in Europe: Portugal (*n* = 5);Sweden (*n* = 1); UK (*n* = 2) Belgium (*n* = 3) Germany (*n* = 1) The Netherlands (*n* = 2) and also, in USA (*n* = 7); Australia (*n* = 4); Canada (*n* = 1) and Japan (*n* = 1). The studies were published between 2004 and 2019, mostly in 2015 or after (*n* = 19).

**Table 1:** Studies included in the review (*n* = 27)

Authors, year, country	Design and JBI	Aim	Number of participants in last measurement point	Instrument(s)
Azermai et al. [36], 2017, Belgium	Pre-post and follow-up intervention with control group JBI = 7	To evaluate the reduce the psychotropic drug use and to improve prescribing practice via intervention	Residents in nursing homes. E: *n* = 118 C: *n* = 275 A: *n* = 393	* Psychotropic Education and Knowledge test [37] * Medication charts
Ballard et al. [38], 2018, United Kingdom	Randomised controlled cluster trial JBI = 9	To evaluate the efficacy of a person-centered care and antipsychotic review on Quality of Life, agitation and antipychotic use in people with dementia living in nursing homes, and to determine its cost.	Older people with dementia in nursing homes. E: *n* = 257 C: *n* = 296 A: *n* = 553	* Health-related quality of life of people with dementia 31 items [39] * Medication charts * Agitation Inventory 29 items [40] * The Neuropsychiatric Inventory 12 domains [41]
Barbosa et al. [42], 2017, Portugal	Experimental, pre-post-test control group design JBI = 9	To assess the effects of a Psycho-educational programme on the quality of direct care workers interactions with residents with dementia.	Direct care workers in aged-care facilities. E: *n* = 27 C: *n* = 29 A: *n* = 56	* Video-recorded sessions coded by Global Behavioral Scale [43]
Barbosa et al. [44], 2016a, Portugal	Experimental, pre-post-test control group design JBI = 9	To assess the effects of a person-centered care based psychoeducational intervention on direct care workers verbal and nonverbal communicative behaviors with residents with dementia during morning care.	Direct care workers in aged-care facilities. E: *n* = 27 C: *n* = 29 A: *n* = 56	* Ethogram for verbal communicative behaviors by framework of Kitwood [45]
Barbosa et al. [46], 2016b, Portugal	Experimental, pre-post-test control group design JBI = 9	To assess the 6-month effects of a person-centered care-based Psychoeducational intervention targeted at direct care workers caring for people with dementia in aged-care facilities.	Direct care workers in aged-care facilities. E: *n* = 24 C: *n* = 29 A: *n* = 53	* Perceived Stress Scale 13 items * Maslach Burnout Inventory 22 items in 3 subscales * Minnesota Satisfaction Questionnaire 20 items in 2 subscales * Ethogram for verbal communicative behaviors by framework of Kitwood [45]
Barbosa et al. [47], 2015, Portugal	Experimental, pre-post-test control group design JBI = 9	To assess the effects of a person-centered care-based psychoeducational intervention to direct care workers stress, burnout, and job satisfaction.	Direct care workers in aged-care facilities. E: *n* = 27 C: *n* = 29 A: *n* = 56	* Perceived Stress Scale 13 items * Maslach Burnout Inventory 22 items in 3 subscales * Minnesota Satisfaction Questionnaire 20 items in 2 subscales * 8 focus group interviews
Boersma et al. [48],2019, Netherlands	Quasi-experimental, pre-post-test control group design JBI = 9	To assess the implementation of the Veder contact method (VCM) in 24-h care.	Professional Caregivers E: *n* = 79 C: *n* = 57 A: *n* = 136 Residents E: *n* = 78 C: *n* = 61 A: *n* = 139	* Quality of Caregivers’ Behavior in dementia care (QCB) 25 items * Approaches to Dementia Questionnaire (ADQ) 19 statements * Quality of implementation of VCM score * QUALIDEM observation tool * INTERACT observation tool * Observation 3 h + 3 h
Boersma et al. [49], 2017, Netherlands	Non-randomized controlled trial JBI = 8	to assess how working with the Veder Contact Method influences the job satisfaction of caregivers’	Caregivers from nursing homes. E: *n* = 75 C: *n* = 36 A: *n* = 111	* Job satisfaction (Leiden Quality of Work Questionnaire) 23 items * Focus group interviews * Interviews of managers
Bökberg et al .[50], 2019, Sweden	Experimental, pre-post-test control group design JBI = 9	To evaluate whether an educational intervention had any effect on staff’s perception of providing person-centered palliative care for older persons in nursing homes.	Staff members in nursing homes. E: *n* = 167 C: *n* = 198 A: *n* = 365	* Person-centered Care Assessment Tool 13 items in 2 subscales [51] * Person-Centered Climate Questionnaire 14 items in 3 subscales [52] (Continued)
Chenoweth et al. [53], 2014, Australia	Cluster-randomized trial JBI = 9	To evaluate differents of effectiveness of Person-centered care (PCC), person-centered environment (PCE) and PCC + PCE interventions to quality of life and agitation for people with dementia in nursing houses.	Residents in nursing homes PCC *n* = 64 PCE *n* = 79 PCC + PCE *n* = 89 C: *n* = 64 A: *n* = 296	* Person-Centered Environment and Care Assessment Tool [54] * Cohen-mansfield Agitation Inventory [40] * Person with Dementia and Quality of life measurement [55] * Emotional Responses in Care * Global Deterioration Scale of Primary Degenarative Dementia * Residents activities of daily living * Cornell Scale for Depression in Dementia * Quality of Interactions Schedule
Chenoweth et al. [56], 2009, Australia	Cluster-randomized trial JBI = 9	To compare effectiveness of Person-centered care (PCC), Dementia Care Mapping (DCM) and usual care and these methods decreases to dementia-compromised behaviors, quality of life, use of psychotropic drugs and injuries. The estimated cost differences between the treatments were also of interest	Residents in nursing homes: PCC *n* = 77 DCM *n* = 95 C: *n* = 64 A: *n* = 236	* Cohen-Mansfield Agitation Inventory [40] * Neuropsychiatric Inventory for the nursing home * Quality of Life measurement * Observation * Recorded information about the drugs * The Therepeutic Environment Screening Survey for Nursing Homes * Interviews * Report of economic analysis
Coleman & Medvene [57], 2013, USA	Quasi-experimental, wait-list control design JBI = 9	To pilot test a multicomponent intervention to increase certified nursin assistans’ awareness of Person-centered care and to establish the feasibility of implementing an intervention involving videotaped biographies of residents and videotapes of resident/ nursin assistans’ caregiving interactions.	Residents / CNAs dyads. E: *n* = 11 dyads C: *n* = 8 dyads A: *n* = 19 dyads	* Video recorded material code by Person-Centered Behavior Inventory and Global Behavior Scale [58] * Relationship between residents and CNAs measured by Resident Satisfaction Index 27- items and Subscale of the Maslach Burnout Inventory “Personal Accomplishment” 8-items [59] and Mutuality Scale 15-items [60]
Cornelison et al. [61], 2019, USA	A multi-arm, pre-post intervention study JBI = 6	To evaluate how nursing homes, perceive their adoption of person-centered care practices across seven domains and how these perceptions change in response to an eductional intervention embedded in a statewide program, Promoting Excellent Alternatives in Kansas nursing homes	Staff teams from nursing homes: E: *n* = (Pre-adopters) 82 C: *n* = (Adopters) 86 A: *n* = 168	* Kansas Culture Change Instrument 68 -items in 7 dimensions
Fossey et al. [62], 2006, United Kingdom	Cluster randomised controlled tria JBI = 8	To assess effectiveness of a training and support intervention for nursing home staff in reducing the proportion of residents with dementia who are prescribed neuroleptics.	Residents in nursing homes E: *n* = 176 C: *n* = 170	* Cohen-Mansfield Agitation Inventory [40] * Dementia Care Mapping (DCM) * Medication charts
Gillis et al. [63], 2019, Belgium	Pre-posttest design, without control group JBI = 6	To test a person-centred team approach for addressing agitated or aggressive behaviour amongst nursing home residents with dementia.	Residents in nursing home *n* = 65	* Neuropsychiatric Inventory – Nursing Home Version (NPI-NH) 12 symptoms * Cohen-Mansfield Agitation Inventory (CMAI) 29 items
Hoeffer et al. [64], 2006, USA	Randomized controlled trial with crossover design JBI = 9	To test the efficacy of two person-centered care-based bathing interventions to improve caregiving behavior during bathing.	Residents E1: *n* = 24; E2: *n* = 22 C: *n* = 23 A: *n* = 69 Nursing assistants E: *n* = 24 C: *n* = 13 A: *n* = 37	* Video-recoded sessions coded by The Caregiver Bathing Behavior Rating Scale * Care Effectiveness Scale two scales; The Confidence Scale 6 items, The Easy Scale 3 items * The Hassless During Bathing Scale eight items
Jeon et al. [65], 2012, Australia	Cluster-randomized trial JBI = 9	To compare effectiveness of person-centered care (PCC), Dementia Care Mapping (DCM) and usual care on staff burnout, well-being, attitudes and reactions toward behavioral disturbances of residents with dementia.	Staff members in nursing homes: E: PCC *n* = 56 DCM *n* = 45 C: *n* = 23 A: *n* = 123	* Maslach Burnout Inventory-Human Services Survey [59] * General Health Questionnaire
Li et al. [66], 2017, USA	Pre-post controlled trial JBI = 7	To test the effects of a Person-Centered Dementia Care intervention on sleep in residents.	Residents in dementia care units. E: *n* = 16 C: *n* = 6 A: *n* = 22	* Actiwatch Spectrum (small device) * Dementia Care Mapping (DCM) * Brief Interview for mental Status * Cumulative Illness Rating Score for Geriatrics
McGilton et al. [67], 2017, Canada	Pre-posttest design, without control group JBI = 7	to examine the effectiveness of individualized communication plans tailored to the needs of residents with dementia.	Residents in nursing home *n* = 12 and nurses *n* = 20 A: *n* = 32	* Mini-Mental State Examination / Color Vision test / Audiometer Test / acuity test * Functional Linguistic Communication Inventory *Cornell Scale for Depression in Dementia 19 items * The Alzheimer Disease-related Quality of Life 40 items * Katz index of ADL 6 items * Communication-Imparment Questionnaire 8 items * Interactional Comfort Survey 5 domains * The Satisfaction Working with Residents with Dementia 21 items * Nursing Care Assessment Scale 28 items * Interviews (focus group and individual) * Observation
Richter et al. [68], 2019, Germany	Cluster-randomised controlled trial JBI = 9	to adopt the person-centred care intervention from UK to German conditions and test its effectiveness	Residents in nursing homes. E: *n* = 493 C: *n* = 660 A: *n* = 1153	* Documents of residents * Quality of Life in Alzheimer’s Disease Scale * Dementia Screening Scale * Cohen-Mansfield Agitation Inventory [40] * Prescriptions of antipsychotics * Safety parameters
Roberts et al. [69], 2015, Australia	Pre-posttest mixed method design, without control group JBI = 6	to describe the development of a composite model of care based person-centered care and report evaluation and results of a pilot project exploring the new model’s feasibility	Staff members *n* = 15 Residents *n* = 16 Next of kin *n* = 15 A: *n* = 46	* Medication charts * Cohen Mansfield Agitation Inventory [40] * Dementia Care Mapping (DCM) * Interviews by using (Resident/Relative Audit Tools and Tool for Understanding Residents Needs as Individual Persons’)
Sloane et al. [70], 2013, USA	Pre-posttest design, without control group JBI = 5	to develop and test a person-centered evidence-based mouth care program in nursing homes	Residents in nursing homes *n* = 88 Nursing assistants *n* = 6 A: *n* = 94	* Plaque Index for Long-Term Care * Gingival Index for Long-Term Care * Denture Plaque Index * Minimum Data Set * Videotaping; Noldus Information Technology Wageningen / Mouth Care Task Completion Form * Nursing home records about resident’s individual health situations
Sloane et al. [71], 2004, USA	Randomized Controlled trial, with two experimental groups and crossover JBI = 9	to evaluate the efficacy two nonpharmacological based person-centered care techniques in reducing agtation, aggression and discomfort in shower and towel bath situations in nursing home residents with dementia	Residents in nursing homes E: *n* = 24/ group A *n* = 25/group B C: *n* = 24 Nursing assistants E: *n* = 24 in groups together C: *n* = 13 A: *n* = 110	* Videotaping * Care Recipient Behavior Assessment * Discomfort Scale for Dementia of an Alzheimer Type 6 items * Hardy Skin Condition Data Form * Skin cultures * Activities of Daily Living * Cumulative Index Rating Scale for Geriatrics * Cohen-Mansfield Agitation Inventory [40]) * Mini-Mental State Examination * Cognition Scale * Recorded Medication data
Sposito et al. [72], 2017, Portugal	Quasi-experimental study, with pre-posttest, without control group JBI = 6	To assess effectiveness of person-centered care, Multisensory Simulation and Motor Simulation intervention in residents’ behavior during the morning care.	Residents in nursing homes: *n* = 45 Direct care Workers in Nursing Homes: *n* = 56 A: *n* = 101	* Mini-Mental State Examination * The Global Deterioration Scale * Video recordings
Wauters et al. [73] 2019, Belgium	Cohort study with cross-sectional observations, with pre-posttest, without control group JBI = 5	To investigate whether the intervention, starting from general intervention template, could be successfully implemented in separate nursing homes, resulting in a decreased prevalence of psychotropic drug users.	Residents in five different nursing homes: *n* = 677	* Medical records and electronic medication charts * Mini-Mental State Examination * Katz Activities of Daily Living (mandatory in Belgium)
Williams et al. [74], 2018, USA	Pre-posttest design without control group JBI = 6	to test four interdisciplinary strategies to measure changes in person-centered communication used by nursing home staff following intervention	Nursing staff *n* = 32 Residents *n* = 49 A: *n* = 81	* Behavioral, psycholonguistic, emotional tone coding of elderspeak communication and content analysis of communication topics
Yasuda & Sakakibara [75], 2017, Japan	Pre-posttest design without control group JBI = 5	To assess the effects of care staff training based on person-centered care and dementia care mapping on the quality of life) of residents with dementia in a nursing home	Residents *n* = 40	* Mini-Mental State Examination * DCM (Dementia Care Mapping) * Barthel Index

*JBI* The Joanna Briggs Institute checklist for Quasi-Experimental Studies, *E* Experimental group, *C* Control groups, *A* All Participants, *PCC* Person-Centered Care, *PCE* Person-Centered Environment, *DCM* Dementia Care Mapping

The design of the studies were RCTs (*n* = 9), experimental designs (*n* = 6), quasi-experimental design (*n* = 2), pretest-posttest designs (*n* = 10). All studies conducted pretests and posttests after the intervention. Four studies were carried out without control group and there were two experimental groups in one RCT design. The quality appraisal, mean and median scores using the JBI checklist, were 7.70 and 9, out of nine respectively.

The study informants were mainly older people (*n* = 11), nurses (*n* = 7) or both together (*n* = 6). In one study nurses responded as a team. In another study nurses and older people formed a dyad. The next of kin of the older people participated in one study together with older people and nurses. In the experimental groups, the mean and mode of the participants were 82 (range 6–677) and 24 respectively. In the control groups the mean and mode were 100 (range 6–660) and 29 respectively.

### Implementation of the continuing education interventions

The pedagogical methods used in the continuing education interventions (Table 2) were contact teaching via seminars, workshops, or team sessions (*n* = 27), usually with the on-site support (*n* = 17). In some studies, a few “key nurses”, attended continuing education (*n* = 5). Within the continuing education sessions digital material such as videos were used (*n* = 8) and in one study simulation and role-playing games.

**Table 2:** Continuing education interventions, pedagogical methods, assessments, and their outcomes

Theme	Content of intervention	Pedagogical methods	Assessments	Outcomes and effectiveness in lens of nurses, older people and next of kin	Source
Medication	-” Awareness-campaign” to the nurses, next of kin and residents - Educational courses given by experts (sleeping problems, old age depression, behavioral problems) - Professional support	- Flyers, posters, and articles in nursing home’s own newspaper - 3 X 2 h - For 10 months two part-time project staff-members offered person-centred professional support to the nurses	Drug use was recorded at baseline, after 10 months and after 22 months in intervention home and only medication data from control nursing home.	P: Quality improvement initiative led to a significant decrease in the use of psychotropic drugs in the intervention group, even after 1-year follow-up. Education only had a limited effect, but education and professional support together had clearer effects in long-term. OP = +	Azermai et al. [36], 2017
	- Orientation for managers, staff teams, WHELD champions and residents - WHELD champions (two/each care home) training - on-site consultation	- 2 days orientation - WHELD champions trained 4 months (1 day per month) - On-site consultation 8 h/month/care home	The quality of life of older people, reduced agitation, general neuropsychiatric symptoms, antipsychotic use, global deterioration, mood, unmet needs, mortality, quality of interactions, pain and cost were assessed at baseline and at 9 months after the intervention.	P: WHELD interventions improved the quality of life of older people S: WHELD reduced agitation, and general neuropsychiatric symptoms, and increased the number of positive interactions between nurses and residents. There were also cost advantages. N = + / OP = +	Ballard et al. [38], 2018
	- Training and supporting by specialists such as a psychologist, occupational therapist or nurse including about the philosophy of person-centered care, positive care planning, awareness of environmental design issues, behavioral models, developing individual interventions, active listening, communication skills, reminiscence techniques and involvement of family careers. - Supervision for the staff members, including systematic consultation, home issues, didactic training, skills modelling	- 10 months training and supporting - Weekly supervision of groups and individual staff members	Daily dose of drugs, agitation, disruptive behaviour, and quality of life were assessed at baseline and after 12 months.	P: The proportion of residents using neuroleptic drugs in research centers was significantly lower than in the control homes. S: There was no effective difference between intervention and control groups in behavioral symptoms, but behavioral symptoms did not increase when less drugs were used. OP = +	Fossey et al. [62], 2006
	- Training and supporting by specialists such as a psychologist, occupational therapist or nurse including about the philosophy of person-centered care, positive care planning, awareness of environmental design issues, behavioral models, developing individual interventions, active listening, communication skills, reminiscence techniques and involvement of family careers. - Supervision for the staff members, including systematic consultation, home issues, didactic training, skills modelling	- Information about the study 60 min. - 2-day workshop - Supporting by experts during the intervention	Antipsychotic drug use, prescriptions of antipsychotics and safety parameters as falls were assessed at baseline and after 3, 6, 9, and 12 months. Quality of life and agitation were assessed at baseline and after 12 months.	P: The intervention did not reduce the use of antipsychotics in nursing homes in Germany, although a reduction was seen in the control group. S: There were not statistically significant differences between the study groups in Quality of Life, agitation, falls, physical restrains or prescriptions of antipsychotics. N = 0	Richter et al. [68], 2019
	- Education about sleeping problems, depression in old age, challenging behaviour. Content of was focused on evidence-based practice, reductions in psychotropic drug use and non-pharmacological alternatives.	- Transition to person-centred care by awareness campaign - Online and printed material - Educational sessions (dose?), recorded and available later online - Assessment of psychotropic drug use (GP/ residents/ nurses /relatives)	Psychotropic drugs use was assessed at baseline and after 12 months.	P: The intervention resulted in a significant decrease in psychotropic drug use among nursing home residents after 12 months. The combination of education, professional support, and the transition towards patient-centred care proved successful in discontinuation of high in-house psychotropic drug like hypnosedative and antidepressant use, except antipsychotics. OP = +	Wauters et al. [73], 2019
Interaction and caring culture	- Education to enhance nurses knowledge and skills concerning person-centered dementia care in eight themes: person-centered care and dementia, the emotional impact of caregiving, communication in dementia, conflict management, challenging behaviours, teamwork, the environment and dementia, motor simulation, problem solving, relaxation and multisensory stimulation. - Individual assisted sessions for nurses by experts - Supportive to improve nurses ability to cope with job-related stress and burnout	- Eight weekly session, 90 min per time - Three days after session, individual sessions with nurses	Direct care workers person-centredness video-recorded and then coded by global behavioral scale were assessed at baseline and after 8 weeks in the end of the intervention.	OC: Person-centered care-based continuing education intervention can be effective during the morning care to residents with dementia. It may increase nurses person-centeredness. Stress support for the test group was not effective. N = +	Barbosa et al. [42], 2017
	- Education to enhance nurses knowledge and skills concerning person-centered dementia care in eight themes: Person-centered care and dementia, the emotional impact of caregiving, communication in dementia, conflict management, challenging behaviours, teamwork, the environment and dementia, motor simulation, problem solving, relaxation and multisensory stimulation - Individual assisted sessions for nurses by experts - Support to improve nurses’ ability to cope with job-related stress and burnout.	- Eight weekly session, 90 min per time - Three days after session, individual sessions with nurses	Direct care workers communicative behaviours with people with dementia were video-recorded and assessed at baseline and 2 weeks after eight-week intervention.	OC: Experimental group had a broader impact with the frequency of all behaviour categories than the control group. Experimental group had more verbal and non-verbal communication than the control group. N = +	Barbosa et al. [44], 2016a
	- Education to enhance nurses knowledge and skills concerning person-centered dementia care in eight themes: Person-centered care and dementia, the emotional impact of caregiving, communication in dementia, conflict management, challenging behaviours, teamwork, the environment and dementia, motor simulation, problem solving, relaxation and multisensory stimulation - Individual assisted sessions for nurses by experts - Supportive to improve nurses’ ability to cope with job-related stress and burnout	- Eight weekly session of 90 min - Three days after session, individual sessions with nurses	Direct care workers’ stress, burnout, job satisfaction, and person-centered communicative behavior with people with dementia were assessed baseline, after eight-week intervention and after 6 months follow-up.	OC: Person-centered care-based continuing education is effective for reducing nurses’ burnout and improving communicative behaviors, up to 6 months after the intervention. Thus, the impact on stress levels sees to deteriorate after 6 months. Continuing education intervention did not influence the job satisfaction. N = +	Barbosa et al. [46], 2016b
	- Functioning of long-term memory in people with dementia, reminiscing and one-to-one contact - Theatrical communication; importance of the” saying goodbye” ritual and relation with the life history of residents - Repeating the information from the first three monthly sessions and discussing the experiences of caregivers and the reactions of residents when applying VCM - Connection is made with the” authentic self” of the caregivers and exercise in how to start up a communication according VCM and related to the life history of residents as described in their care plan.	- Three monthly training sessions of 3 h - Three on-the-job coaching training sessions (1 h per session) before training sessions. - Two 3 h follow-up training sessions	Attitude of professional caregivers and self-rated ability to work with a care plan were assessed by measurements at baseline and at the end of the one-year intervention. The communicative behaviour of caregivers and residents’ behaviour were observed at baseline and at the end of the intervention.	OC: Significant improvements in caregivers’ communicative behavior and some aspects of residents’ behavior and quality of life were found on the experimental wards with a high implementation score. No significant differences were found between the groups in caregivers’ attitudes, residents’ care plans, or mood. N = + / 0 OP = + / 0	Boersma et al. [48], 2019
	- Education including lectures on person-centered care, communication and relationships.	- 4 X 1 h sessions - Demonstration videos, homeworks, discussion, using an interaction worksheet.	Nursing assistants’ awareness of Person-centered care and interactions with residents and nursing assistants were assessed at baseline, 6 weeks after intervention and 7 weeks after that.	OC: Theory-driven person-centered training intervention for nurses could be developed and implemented in Nursing Homes. Residents reported a closeness of relationship with the CNA. Both CNAs and residents reported increased satisfaction in their relationship after the training period. N = + OP = +	Coleman & Medvene [57], 2013
	**Content of intervention**	**Pedagogical methods**	**Assessments**	**Results and effectiveness in lens of nurses, older people and next of kin**	**Source**
	- Education by mentor homes	- Workbook - DVDs	Nursing homes perceive their adoption of person-centered care practices were assessed at baseline and after one-year education.	OC: Pre-adopters had lower scores 1 year after the education. This may be influenced the conceptualizing of person-centered care during the education. Better understanding of the concept of person-centered care can improve the rate of adoption. Education and training are important when changing the caring culture. The nurses role in the perception of person-centered care practices is important. N = 0	Cornelison et al. [61], 2019
	- Therapeutic touch - Music therapy - Individualized meaningful activity	- 2 h training session	Non-pharmacological intervention based on the resident’s underlying needs was assessed at baseline and 3 days after the last session in 2 months intervention time.	OC: The frequency of aggression, loss of decorum, depression and the severity of aggression decreased for all three interventions. Person-centred team-based approach is effective to reduce agitated or aggressive behaviour amongst nursing home residents. OP = +	Gillis et al. [63] 2019
	- Individual communication plans - Dementia care workshop - Support system, for nurses	- 4-h workshop - Support by experts	Residents mood and daily functioning were assessed at baseline and 10 weeks after the care providers were instructed in using the communication plans. Care providers’ attitudes, satisfaction, and burden were assessed at baseline and 10 weeks following the workshop.	OC: Individually tailored resident interventions may improve the quality of life of residents with dementia. Also positive effects on care providers’ mood and burden were measured. N = + OP = +	McGilton et al. [67], 2017
	- Information about the project and the engagement of doctors, nurses, managers and the older people’s next of kin to the project - Education about the dementia care and Montessori activity training - Support system - Environment changes	- Four days education - One day / month consultation on nursing home - Support by phone and email	ABLE model was assessed at baseline and 12–14 months after the intervention.	OC: Significant behavior changes were evident among residents. Staff reported increased knowledge about meeting the needs of people with dementia and organizational culture change experiences. Next of kin were satisfied for the changes. N = + NK = + OP = +	Roberts et al. [69], 2015
	- Nurses and residents’ dyads were video recorded before and after the intervention - The first session introduced effective and ineffective communication; video vignettes were used - The second session focused on elderspeak and its identification and negative effects, video recordings used to provide examples. - The third session taught positive communication strategies, participants critiqued videos and corrected transcripts eliminating elderspeak	- 3 X 1 h group session - Videos	Changing Talk communication intervention was assessed by collected video recordings at baseline, immediately after the 3 weeks intervention and at three-month follow-up.	OC: Post-intervention improvements in communication occurred for each measure; however, the changes were statistically significant only for behavioral and psycholinguistic measures. Methods and results for each communication measure were compared. N = + OP = +	Williams et al. [74], 2018
	- Knowledge and skill concerning of person-centered care. - Information on dementia, verbal and non-verbal communication strategies, multisensory stimulation types.	- 8 weekly group sessions (90 min/session) by experts - Group discussions, simulations, homework exercises, role-playings and brainstorming	Multisensory and motor stimulation intervention effects were assessed at baseline and after the eight-weeks intervention through video-recordings.	P: Intervention seems to increase the frequency of engagement in the morning tasks. S: Residents’ frequency of closing their eyes decreased. They were less sad, smiled more and engaged in verbal communication more after the intervention increased. OP = +	Sposito et al. [72], 2017
Nurses’ job satisfaction	- Education to enhance nurses knowledge and skills concerning person-centered dementia care in eight themes: Person-centered care and dementia, emotional impact of caregiving, communication in dementia, conflict management, challenging behaviours, teamwork, environment and dementia, motor simulation, problem solving, relaxation and multisensory stimulation. - Individual assisted sessions for nurses by experts - Supportive to improve nurses’ ability to cope with job-related stress and burnout	- Eight weekly session, 90 min per time - Three days after session, individual sessions with nurses	Direct care workers stress, burnout and job satisfaction were assessed at 2 weeks before and 2 weeks after the intervention.	OC: Continuing education intervention has a significant positive effect on nurses’ emotional exhaustion. According to the qualitative data, the experimental group perceived enhanced cohesion, emotional management, and self-care awareness. This can reduce nurses’ burnout. N = +	Barbosa et al. [47], 2015
	- Knowledge transfer and skills training in focus on meetings - on-the-job coaching included behavioral observation and direct feedback	- 5 X 3-h team meetings - 3 × 3-h on-the-job coaching sessions	Veder Contact Methods effects to the caregivers’ job satisfaction was assessed at baseline and after the 9 months intervention.	OC: The intervention had no significant effect for job satisfaction using the quantitative data findings. The qualitative data findings indicate that intervention has positive influence on the daily work performances of nursing home caregivers. N = 0 / +	Boersma et al. [49], 2017
	- Person centered care group: two selected nurses per site engaged in off-site person-centered care education and after that person-centered care expert visited two full days on-site to assist these nurses to develop individual residents care plans and to implement person-centered care - Dementia Care Mapping group: two selected nurses per site engaged in off-site dementia care mapping and person-centered care education and after that experts worked alongside them to conduct dementia care mapping for all participating residents.	- Person-centered care: 2-day education off-site and 2-day on-site guide to implement person-centered care. Support by phone for 4 months. - Dementia care mapping: 3-day education off-site and alongside worked with selected nurses after the education. Support by phone for 4 months.	Staff burnout, general well-being, attitudes, and reactions towards residents behavioural disturbances, perceived managerial support and quality of care interactions were assessed at baseline, immediately after intervention and at 4 months’ follow-up.	P: Dementia care mapping was more effective than person-centered care to reduce the staff members’ job-related burnout and emotional exhaustion. The support of managers is important. Without the managers support the influence of intervention is weaker and lasts less time. S: There were no significant differences in terms of staff attitudes and reactions towards behavioral disturbances and care quality. N = +	Jeon et al. [65], 2012
Nursing activities	- Knowledge-based seminar material based on two Swedish national documants about the key principles of palliative care. - The Eductional booklet used as study material with nurses had five themes: palliative care and dignified care, next of kin, existence and dying, symptom relief, collaborative care.	- 5 X 2 h education seminars	Person-centeredness education intervention effectiveness on the staff’s perception of providing person-centred palliative care for older people in nursing homes was assessed at baseline and 3 months after the 6 months intervention.	OC: The intervention was not effective, because results showed no improvement in any outcomes. The only perceived improvement area in person-centered care was the managers’ and organization’s support of the staff’s everyday work to maintain person-centered care. N = 0	Bökberg et al. [50], 2019
	- Education about the person-centered approach to showering and and towel baths focusing on residents’ needs, the accommodation of residents’ preferences, attending to the relationship and interaction with residents, using effective communication and interpersonal skills, adapting the physical environment and bathing procedures to decrease distress and discomfort.	- 2 × 6 weeks training periods including didactic sessions, reviewed videotaped material, coaching in bathing situations by clinical nurse specialist	Effects of two bathing interventions on caregiving were assessed at baseline, end of the first 6 weeks intervention, and end of the next 6 weeks intervention also.	OC: Bathing interventions improved gentleness, verbal support, confidence, and ease, but not reduce the hassles. N = + OP = +	Hoeffer et al. [64], 2006
	- Developed from Kitwood’s model of Dementia and framework for person-centered care: understanding dementia (the person and disease), being with the person who has dementia, making a difference in the life of the cognitively impaired.	- One 2 h and two 3-h lectures with learning exercises and role playing. - On-site training twice per week × 4 weeks (total 16 h) - 4-h dementia care mapping session - On-the-job practicing - Consultation	Sleeping of assisted living residents with dementia was assessed 3 days at baseline and 3 days after the intervention.	P: Staff education intervention may have effective for improving the sleep of residents with dementia. In the intervention group residents had significantly more night-time sleep and less daytime sleep than in control group. OP = +	Li et al. [66], 2017
	- Oral pathology, dementia care, individualized care planning and skills training	- Seminars - On-site training daily for 2 weeks by dental hygienist and geriatric psychologist - Consultation.	Residents’ oral hygiene were assessed at baseline via videotaped performing mouth care by CNAs and during the 6 weeks intervention by measurements and video recording by CNAs.	P: The intervention had a significant effect on the residents’ oral hygiene outcomes as Plaque Index and Gingival Index. S: after the intervention mouth care was more thorough, took more time and consistency of care appeared to be more important for natural teeth than dentures. OP = +	Sloane et al. [70], 2013
	- Person-centered bathing focused on resident comfort and preferences, viewed behavioral symptoms as expressions of unmet needs, employed communication techniques appropriate for the resident’s disease severity, applied problem-solving approaches to identify causes and potential solutions and regulated the physical environment to maximize resident comfort.	- 2 × 6 weeks training periods including didactic sessions, reviewed videotaped material, coaching in bathing situations by clinical nurse specialist	Nonpharmacological techniques in reducing agitation, aggression and discomfort in nursing home residents with dementia in bathing situations and skin condition were assessed at baseline and end of both intervention periods.	P: Person-centered showering and the towel bath constitute safe, effective methods of reducing agitation, aggression, and discomfort during bathing of people with dementia. S: Average bath duration increased significantly in showering. Neither intervention resulted in fewer body parts being bathed, both improved skin condition. N = + OP = +	Sloane et al. [71], 2004
Older peoples’ quality of life	- Person-centered care: residents’ feelings when agitated, interacting with residents in a person-centered way and using Person-centered care planning to meet the residents’ psychosocial needs. - Person-centered education: improvements to the safety accessibility and utility of outdoor spaces, provision of a greater variety of social spaces and using colour and objects for wayfinding and to improve feelings of familiarity.	- Person-centered care: 32 h off-site education for” Key” nurses and on-site supervision (range 2–16 h) and support by phone.	Difference effects of PCC and PCE interventions were assessed at baseline, after 4 months intervention and at 8 months follow-up.	P: Person-centered care and person-centered education interventions together did not seem to improve the quality of life or reduce agitation but improved emotional responses to care. Depression scores did not change in any of the groups. S: Education interventions together seems to improve in care interaction quality. OP = 0	Chenoweth et al. [53], 2014
	- An understanding that behaviour is a form of communication. Nurses need to recognize that feelings persist despite cognitive impairment and acknowledge feelings during social interactions. Nurses should focus on the unique way that residents express feelings and needs to change usual care.	- Person-centered care: 2-day training sessions for two nurses per site - Two visits per site - Dementia care mapping: 2 X 6 h training for two nurses per site - Both: on-site assisting to implement dementia care mapping/person-centered care for residents and support by phone	Comparison of person-centred care, dementia care mapping and usual care effects to agitation and psychiatric symptoms were assessed at baseline, after 4 months intervention and at 4 months of follow-up.	P: Both person-centered care and dementia care mapping seems to reduce agitation. S: Person-centered care was less safe than dementia care mapping, because falls happened more in person-centered care groups. Person-centered care had more positive social and care interactions than other groups. Interventions did not have any influence on the level of medication use. Person-centered care intervention costs were lower than dementia care mapping. OP = +	Chenoweth et al. [56], 2009
	- The concept of dementia; how to respond to dementia; the concept of person-centered care; and specific methods of interacting with residents with dementia. - Discussions, based on the dementia care mapping results, focused on the following: consideration of the behaviors of residents with dementia; respecting residents during interactions; and improving future care processes.	- 3 X 60–90 min training sessions	Effects of staff training on person-centred care and dementia care mapping on quality of life of residents with dementia were assessed at 1 month before baseline, at baseline and last after the intervention.	OC: Person-centered care-based staff training, and dementia care mapping could effectively improve the quality of life of residents with dementia. OP = +	Yasuda & Sakakibara [75], 2017

*N* Nurses, *OP* Older People, *NK* Next of Kin, *+* positive effect, *0* non effect, *P* Primary outcome, *S* Secondary outcome, *OC* Outcome, if the authors did not discriminate between primary and secondary outcomes

The continuing education interventions were categorized into five themes: (1) medication (*n* = 5); (2) interaction and caring culture (*n* = 11); (3) nurses’ job satisfaction (*n* = 3); (4) nursing activities (*n* = 5); and (5) older people’s quality of life (*n* = 3). In the medication themed interventions, the typical goal was to reduce the number of medications older people took and to learn to use person-centered care to reduce behavioral issues that disturbed other residents. The aim of the interaction and care culture themed interventions was to increase positive communication between nurses and the older people and to influence the caring culture, making it more person-centered. Some continuing education interventions focused on increasing nurses’ job satisfaction. Nursing activities themed interventions were aimed at influencing residents’ daily routines and activities.

### Outcomes of the continuing education interventions

Most of the continuing education interventions about person-centered care had positive effects (Table 2) but four studies indicated that the interventions did not have the any effect [50, 53, 61, 68].

Medication themed intervention outcomes described a significant decrease in use of psychotropic drugs [36, 73]. The proportion of residents using neuroleptic drugs in research centers was significantly lower than in control homes [62] which reduced costs [38]. Continuing education intervention influence on the staff and older people’s behavior varied. The interventions had no effect on residents’ behavioral symptoms such as agitation and disruption in one study, but these symptoms also did not increase with fewer medications [62]. In another study, continuing education intervention did reduce residents’ agitation, their general neuropsychiatric symptoms and increased their positive interactions between nurses and residents [38].

Interaction and caring culture themed continuing education interventions had a positive influence on: nurses’ person-centeredness [42]; verbal communication [44, 48, 71]; non-verbal communication [44]; increased direct gaze duration between residents and nurses; reduced sadness and increased smiling [72]. In one study, both nurses and residents reported increased satisfaction in their relationship after the continuing education intervention and residents reported a closer relationship with nurses [57]. Nurses reported increased knowledge about meeting the needs of people with dementia and organizational culture change experiences and next of kin were also satisfied with these changes and quality of care [69]. Also, positive effects on care providers’ mood, burden [67] and residents’ behaviour were reported [63].

Nurses’ job satisfaction themed continuing education interventions seemed to positively influence nurse’s emotional exhaustion, emotional management, self-care awareness and enhanced cohesion within the group, which can reduce nurses burnout [47]. One study had no significant effect on job satisfaction based on quantitative data analysis but included details of a qualitative analysis which showed a positive impact of the intervention on daily work performance [49]. It was suggested that without managerial support, the influence of the continuing education intervention would not be as strong and would be less likely to be established in the workplace [65].

Nursing activities themed continuing education interventions demonstrated the abstract nature of person-centered care [14]. In one study, the continuing education intervention, delivered using a digital device, reported improved sleep for residents with dementia. The experimental group of residents had significantly more nighttime sleep (*p* = 0.03) and less daytime sleep (*p* = 0.01) in the post test [66]. Nursing activities such as bathing [64]; reduced agitation, aggression, and discomfort in persons with dementia [71] and oral hygiene [70] had a positive influence.

The quality of life of older people themed continuing education interventions outcomes varied. In some studies, there was no effect on the quality of residents life [53] but in another study the intervention has got effectiveness to the residents’ quality of life [75]. On these quality-of-life continuing education interventions is usually compared different education methods as person-centered care and Dementia Care Mapping [56].

### Instruments used in continuing education interventions

Although all reviewed studies included person-centered care within the educational intervention, only one study [50] used a validated person-centered care instrument to measure person-centered care outcomes. The Person-Centered Environment and Care Assessment Tool was also used, but its validity is difficult to evaluate as it is within an unpublished PhD thesis [54]. The most commonly used quantitative instruments were, the Cohen-Mansfield Agitation Inventory [40] (*n* = 7), and the Maslach Burnout Inventory [59] (*n* = 4) (Table 1).

## Discussion

This study aimed to analyze and synthesize the existing research literature about person-centered care-based continuing educational interventions for nurses working in long-term care settings for older people. The analysis revealed the focus of this continuing education is on: older people’s medication; the interaction and caring culture; nurses’ job satisfaction; nursing activities and older people’s quality of life. Much of the delivery of this continuing educational training used behaviorist, using pedagogical methods such as lectures and seminars. The method of delivery of education can have an influence on the effectiveness of the intervention [38, 42], but it may be useful to use more learner-centered approaches to improve outcomes in future research [76]. The outcomes of the continuing education interventions of person-centered care were largely collected e.g. from nurses [42, 48, 50] observation of older people or e.g. from older people’s documents [36, 38, 68]. It may be useful, if data on outcomes were also routinely collected from older people and their next of kin [77].

The content of the continuing education activities included person-centered care elements that were designed to influence the interactions between the residents and nurses, and through this, the care environment. However, the influence of continuing education on person-centered care was measured in only one study [50]. Differences in the type of design, outcomes, number of participants, and duration of continuing education intervention hindered study comparisons and generalizations. Moreover, a range of methodological weakness made it difficult to provide any conclusive indication about the effectiveness of these approaches. This heterogeneity of the background theories and measurements of the continuing education interventions used in the studies reviewed, gives the impression of conceptual imprecision. Other researchers support this view [26] thought there is evidence that theory-based educational interventions are effective [78]. Although person-centered care is an abstract concept and so difficult to measure, the creation of a stronger argument for its use requires more rigorous research including the wider use of valid, person-centered care measurement instruments.

Pedagogical methods were at the core of the effectiveness of the continuing education interventions. Some of the studies educated “key nurses,” leaving these nurses to help other nurses learn to implement change in their workplace [50, 53, 56, 61]. There are some risks associated with using “key nurses” in this way, as the process of implementing person centered care requires an understanding of the origin and content of the concepts alongside the practical uses in care situations. The work environment, level of job satisfaction, managerial approaches and the personalities of nurses can help or hinder the work of these “key nurses”, who will require stronger managerial support, encouraging flexible working practices and the involvement of nurses in care decisions [53]. Earlier studies have reported that the collective competence of a team is greater than one persons’ competence [21]. Studies in this review using “key nurses” to introduce person-centered care were not effective in developing the collective competence of the nursing teams directly, limiting the possible benefits of the education e.g. [50].

When introducing person-centered care, pedagogical methods such as face-to-face teaching, though important, were not seen to be sufficient generally [36] and most of the interventions in this review used additional on-site support and consultation e.g. [42, 48, 68, 70] video material e.g. [57, 61, 74] brainstorming [72] and role-playing [68, 72] to achieve their preferred outcomes. Additionally, more student-centered approaches such as simulation-based nursing education interventions have been shown to improve critical and creative thinking [79] though simulation was only used in one reviewed study [72].

Digitalization as part a pedagogical method can support learning activities [80, 81]. Appropriate applications including learner-centered approaches, improve communication between students and educators and enable a collaborative learning environment [82]. However, use of these digital platforms have strengths and weaknesses. It has been reported that Moodle promotes professionalism, ethical behavior and develops critical thinking, but the use of other participatory web-based platforms, including social media platforms may suffer from credibility of information and are open to student misinterpretation [83]. The choice of appropriate platform requires some competence [80].

In this review, after the continuing education, nurses: increased the number of positive interactions with residents [38], improved person-centered care delivery [42], communication [44], and satisfaction in relationship with older people [46, 57], and increased their knowledge about meeting the needs of people with dementia [69]. Other improvements delivered by the outcomes of the reviewed studies were: a reduction in the use of medicines [36, 73]; improved behavioral symptoms [38, 56, 62, 69, 71] and relationships with nurses [57, 64, 72, 74] improved the quality of life for the older people [48, 67, 75]; and increased the number of support for daily activities [66, 70]. These results demonstrate the usefulness of research of continuing education interventions, especially when these changes have been brought about in work practice: reduce nurses burnout [46, 47, 65] have positive effects on nurses mood and burden [67]; and influence organizational culture, changing nurses experiences for the better [48, 69]. This general increase in the quality of care and care environment after suitable continuing education is also supported by other evidence [17].

The data that informed the research came from: residents’ medication charts; nurse-completed questionnaires; video recordings and from observation. In only one study did researchers collect data from the next of kin, about person-centered care interventions [69]. This is disappointing as the opinions of the older people and their next of kin on the results of the continuing education intervention might facilitate a better understanding of the interventions that meet their needs. The lack of old people’s and their next of kin views and perceptions need to be addressed in future research.

Most of the interventions used, produced the positive outcomes described above. However, in some studies the sample sizes were quite small and not power analyzed e.g. [42, 57, 75]. In other studies the content or delivery of the intervention may not have been appropriate [50, 61, 68]. Many other weaknesses were described in the studies reviewed. Additionally, the drop-out rate from the research may have been a major difficulty [48, 50]. This drop-out occurs, for example through the mortality of the residents, and nursing staff turnover [84, 85]. Four studies did not report any positive outcomes from the continuing education interventions [50, 55, 61, 68]. There were quite several studies that did not follow the reporting guidelines, in which case the primary and secondary measures and outcomes were not clearly mentioned [42, 44, 46–50, 57, 61, 63, 64, 67, 69, 74, 75].

This review has identified and analyzed the available continuing education interventions about person-centered nursing in studies from many different countries. Overall, the studies are difficult to evaluate thoroughly often lacking rigorous conceptual and theory bases. This situation could be improved by careful design, choice of research settings, and strict protocols designed to provide robust evidence about the effectiveness of continuing education interventions.

### Strengths and limitations

This review has some strengths. Firstly, this review focused on all research-based continuing education interventions about person-centered care, targeting nurses in older people’s long-term care. The review sought literature from five relevant databases without time limits to provide the best opportunity to find as much of the relevant literature as possible. Using the inclusion criteria, the review considered all available experimental designs, randomized controlled trials alongside those with no control group. This approach facilitated the capture of the widest variety of continuing education intervention studies in this field [86, 87].

The computerized search from databases was conducted by one researcher with the support of an information specialist and then determined by research group.

Secondly, we used the whole research team to assist with study selection, reducing selection bias through discussion towards consensus. For example, in step one (Fig. 1) the citations were evaluated by two researchers first independently and then together. Where consensus was not found a third opinion was sought from another member of the research group.

The limitations of the review relate to the quality of the available studies, limited language only in English and unpublished studies. The research quality appraisal was challenging, because of the limitations of the study designs or number of participants, even though the quality score averaged 7.70 out of 9 [34]. Statistically, the meta-analysis was not applicable because the interventions were different in content and the outcome variables were not comparable [86].

## Conclusion

This review enhances the understanding about person-centered care-based continuing education interventions for nurses working in long-term care settings for older people. Firstly, we identified five themes describing the contents of this type of continuing education. However, concept person-centered care is used in quite different ways and the use of stronger theory-based interventions which can be measured with validated person-centered care instruments is still required. Secondly, we identified the pedagogical methods which were used in these continuing education interventions about person-centered care. Pedagogical methods are quite traditional and could be enhanced using more learner-centered approaches such as appropriate simulation, digital platforms and social media which were not used sufficiently in the reviewed studies. Thirdly, we found that the evaluation of the results of continuing education interventions were mainly conducted from a nurse’s perspective, through for example, medication charts or by qualitative methods such as observation. The perspective of the older person and their next of kin was not evident and should be taken into consideration in future research.

Continuing educational interventions for nurses working in long-term care settings for older people need to be further developed to strengthen nurse’s competence in person-centered care. The positive outcomes in the five themes identified in this review improve the quality of older people’s long-term care. More empirical research-based continuing education interventions are needed, that include a wider set of learner-centered pedagogical methods with measurable outcomes which consider the opinions of older people and their next of kin. These measurable outcomes should be quantified using validated instruments. These developments are important for the quality of care delivery, the quality of life of older people in care and nurses job satisfaction.

## Data Availability

The dataset from this study is available from the corresponding author upon reasonable request.

## Abbreviations

CINAHL: Cumulative index to nursing and allied health literature
PsycINFO: A world-class resource for abstracts and citations of behavioral and social science research
ERIC: Education resources information center
PRISMA: Preferred reporting items for systematic reviews and meta-analysis
RCT: Randomized controlled trial
CCT: Controlled clinical trial
JBI: The Joanna Briggs Institute checklist for quasi-experimental studies
N: Nurse
NK: Next of kin
OP: Older people
+: A positive effect of the intervention
0: Non effect of the intervention
P: Primary outcome
S: Secondary outcome
OC: Outcome
E: Experimental group
C: Control group
A: All participants
PCC: Person-centered care
PCE: Person-centered environment
DCM: Dementia care mapping
